# Palivizumab Prophylaxis among Infants at Increased Risk of Hospitalization due to Respiratory Syncytial Virus Infection in UAE: A Hospital-Based Study

**DOI:** 10.1155/2019/2986286

**Published:** 2019-12-01

**Authors:** M. Elhalik, K. El-Atawi, S. K. Dash, A. Faquih, A. D. Satyan, N. Gourshettiwar, A. Khan, S. Varughese, A. Ramesh, E. Khamis

**Affiliations:** Neonatal Intensive Care Unit, Latifa Women & Children Hospital, Dubai Health Authority, Dubai, UAE

## Abstract

**Background:**

Respiratory syncytial virus (RSV) represents a significant public health burden and the leading cause of lower respiratory tract infections globally, and it is the major cause of hospitalization during the winter. We aimed to evaluate the effectiveness of palivizumab prophylaxis to reduce the hospitalization in children at high risk of RSV infection.

**Methods:**

We performed a retrospective observational single-arm hospital-based study including five RSV seasons (September to March) from 2012 to 2017. We retrospectively included premature infants born at less than 35 weeks of gestation with chronic lungs disease or hemodynamic significant congenital heart disease for palivizumab prophylaxis against RSV infection according to the criteria presented.

**Results:**

A total of 925 children were enrolled in the study over the five RSV seasons. Of them, 410 (44.3%) infants born at <32 weeks of gestation and 515 (55.6%) infants born at 32–35 weeks of gestation with mean (±SD) birth weight of 1104.8 ± 402.85 and 1842.5 ± 377.5, respectively. The compliance with the course of palivizumab was reported in 841 (90.9%) children. Of them, about 75 (8.9%) hospitalized children were reported, and 17 (2.02%) RSV positive children were detected. Hospitalization due to RSV infection was decreased from 9.23% in the 2012-2013 season to 0.67% in the 2016-2017 season.

**Conclusion:**

This study demonstrated that palivizumab prophylaxis in children at high risk of developing RSV infection was effective in reducing the risk of hospitalization with a high compliance rate over the five RSV seasons.

## 1. Introduction

Respiratory syncytial virus (RSV) is a single-stranded, negative-sense ribonucleic acid (RNA) virus and belongs to the genus Orthopneumovirus, family Pneumoviridae, and order Mononegavirales [1]. RSV was divided into two antigenic subgroups, A and B, based on the gene sequence variability of the second hypervariable region located on the distal third region of the G gene [2].

Peak RSV timing is highly focused on winter months in temperate locations of the Northern Hemisphere, with a mode in February [3]. RSV epidemics start in the South moving to the North. Results from a global seasonality of RSV study showed that RSV waves in Southern Hemisphere countries start between March and June while, in the Northern Hemisphere, between September and December [4].

Acute lower respiratory tract infection (LRTI) remains one of the leading causes of morbidity and mortality in children less than five years of age globally. The most common viral pathogen identified in children with LRTI is the human respiratory syncytial virus (RSV) [5]. RSV hospitalization rates were highest among premature infants less than one year (63.9 per 1000) [6].

In 2015, there was about 33.1 million episodes of RSV in children with LRTI globally, which lead to 3.2 million (2.7–3.8) hospital admissions and 59600 (48000–74500) in-hospital deaths in children younger than five years. It also reported that the overall mortality due to RSV in children with LRTI could be more than 118200 (94,600–149,400) [7]. About 45% of the hospitalization (1.4 million) and death (27,300) occurred in young infants <6 months of age.

In the Middle East, the number of children admitted with RSV diseases from developing countries in 2005 was more than double than that estimated in 1986, and the incidence of RSV induced acute LRTI was twice than that of developed countries [8]. A review of RSV infections in the Middle East shows that RSV infections occurred in the winter season peaking around January as in other parts of the world. In Saudi Arabia, RSV hospitalizations showed that the majority of cases were due to bronchopneumonia, prematurity, and lung/heart disorders [9]. While in Kuwait, RSV was the commonest viral infections identified in 52% cases of bronchiolitis, 29% of pneumonia, and 51% of croup [10]. Prevalence of RSV in LRTI equals 28.6% among children less six years in the United Arab Emirates (UAE) [11].

Immunoprophylactic drugs for RSV were developed after the epidemiologic studies, suggesting that infants with high RSV titers of maternally acquired RSV-neutralizing antibody develop less severe RSV disease [12]. Preclinical studies demonstrated that higher immunoglobulin G antibodies against the RSV F glycoprotein correlated with a decreased incidence of severe RSV infection and reinfection [13].

Palivizumab was licensed in June 1998 by the Food and Drug Administration for the reduction of serious lower respiratory tract infection caused by RSV in children at increased risk of severe disease [14]. Palivizumab prophylaxis resulted in significant reductions in the number of RSV-related hospitalizations including the number of RSV-related hospital days with a moderate to severe lower respiratory tract illness, the number of RSV-related hospital days with increased supplemental oxygen, and the incidence of admission to intensive care units [15, 16].

The IMpact-RSV Study in 1996-1997 was the first to investigate the effect of Palivizumab to reduce the hospitalization from respiratory syncytial virus infection in high-risk infants. It revealed an overall reduction in RSV hospitalization rate from 10.6% among placebo recipients to 4.8% among children who received palivizumab prophylaxis, which is 55% reduction [17]. In a descriptive single-centered study performed in Qatar across three RSV seasons (2009 to 2012), the compliance rate of palivizumab immunoprophylaxis was 85.7%, and the cumulative RSV hospitalization rate was 1.9%. They also observed that increase in the compliance rate is directly proportional to a corresponding decrease in RSV-LRTI-related hospitalization, from 3.7% to 1.7% [18].

Data about RSV-related LRTI and palivizumab immunoprophylaxis are scarce in the United Arab Emirates (UAE). This study is performed to determine the effect of RSV immunoprophylaxis on the incidence of RSV-LRTI, over a 5-year period in the UAE.

## 2. Materials and Methods

### 2.1. Study Design and Procedures

This is a retrospective observational study and is conducted at the department of pediatrics of Latifa Women and Children Hospital (LWCH), Dubai, UAE. We retrieved all clinical information of the patients from their documented charts and medical records. The study population involved all at-risk infants discharged from the neonatal intensive care unit (NICU) and are eligible for RSV immunoprophylaxis. Study duration was five consecutive RSV season, between September 2012 and March 2017. We followed the enrolled children up to two years of age.

As per the prevailing pattern in the middle east, RSV season begins during winter months, from September or October up to March of every year. Majority of admission related to RSV occurred during the earlier epidemic months.

The beginning of the RSV season was determined if more than two consecutive patients with positive RSV infections were admitted to the hospital, while the end of the season was determined if less than one patient with positive RSV infection per week for two consecutive weeks was admitted to the hospital.

Nasopharyngeal secretions from at-risk hospitalized infants were collected. RSV infection was confirmed by the Multiplex real-time polymerase chain reaction assay (RT-PCR) using fast tract diagnostics. Samples were collected from nasopharyngeal aspirates from each hospitalized infant.

The dose of palivizumab used was 15 mg/kg/dose intramuscular injection every month for a total of 3–5 doses over the RSV season, according to the starting month of the first dose and eligibility criteria. Baseline data on demographics, the subject's neonatal course, and details of palivizumab administration were collected, respectively, from the patient's record.

This study was approved by Dubai scientific research ethics committee (DSREC), Dubai Health Authority (DSREC-11/2018_3), and informed consent from the parent for each participant was obtained.

### 2.2. Study Population

We included premature infants who were <35 weeks of gestational age at birth and fulfilled the inclusion criteria listed in Table 1. In case of a high-risk infant of a multiple birthset approved for the season, the siblings in the same set are also eligible for same prophylaxis. We followed the American Academy of Pediatrics recommendation for palivizumab immunoprophylaxis (Modified recommendation 2009) [19, 20].

We excluded infants having congenital heart disease (CHD) who were not hemodynamically significant, surgically corrected CHD, and not requiring medication for congestive cardiac failure. Also, infants having life-threating congenital or genetic anomalies were excluded.

### 2.3. Study Objectives and Data Collection

The primary outcome measure of this study was to determine the effectiveness of RSV immunoprophylaxis on the rate of severe RSV-LRTI and related hospitalization. However, the secondary outcome measures were to determine the compliance (received prophylaxis as per guideline), the incidence of RSV positivity, age at the first dose of palivizumab, the need for pediatric intensive care unit admission, and the length of hospital stay.

Baseline demographic characteristics, medical history (e.g., gestational age, age at the 1^st^ dose of prophylaxis, birth weight, multiple birth status, and presence of risk factors like CHD and CLD), and social history (e.g., mother's age, sibling histories, tobacco smoke exposure, and childcare center exposure) were obtained from the medical records of the hospitals using a standardized questionnaires. The parents were queried in Arabic/English, and the information was recorded. Retrospectively, medical records were searched, and required demographic, epidemiologic, and clinical data were collected systematically.

### 2.4. Statistical Analysis

The data were analyzed using SPSS version 25 software. All categorical variables were presented in frequency and percentage, whereas the numerical variables were presented with descriptive statistics (median and IQR). To compare the baseline data and the outcome variables on a continuous scale, 2 sample *t*-tests or Mann–Whitney test were used as appropriate. To compare the baseline and outcome variables on the nominal type of data, the Fisher exact test or chi-square was used as appropriate. Effect of independent variables, i.e., gestational age, birth weight, CHD, CLD, and smoke exposure (2 or more smokers in the household), on the primary outcome was evaluated in a logistic regression model. The odds ratio (OR) and 95% confidence interval (95% CI) were reported. A value of *p* < 0.05 was considered statistically significant.

## 3. Results

A total of 925 eligible children were enrolled in the study across five RSV seasons (September to March) from 2012 to 2017. Most of the admission related to RSV occurred during the earlier epidemic months and within one to two doses of RSV immunoprophylaxis. In the 2012-2013 season, about 66.70% (4 out of 6) of the admission occurred during November and December. The subsequent seasons also demonstrated a similar pattern, where more than half of the RSV infection-related hospitalization occurred during November and December.

Demographics, medical history, and family history are presented in Table 2. Out of the 925 enrolled infants, 44.30% (*n* = 410) were born at <32 weeks of gestation with median (IQR) gestational age of 30.28 (4.1) weeks, while 55.6% (*n* = 515) infants were born at 32–35 weeks of gestation with a median (IQR) gestational age of 33.04 (4.2) weeks at birth. The median (IQR) birth weight of the included babies was 1100.8 (383.8) grams and 1820.5 (397) grams for babies born at <32 weeks and 32–35 weeks, respectively.

For all enrolled babies, 54% (*n* = 501) of them were female. The included boys were younger and had lower birth weight at birth compared to girls. Chronic lung disease was diagnosed in 19.4% (*n* = 180) of the included infants who had CLD, which were more in babies with gestational age <32 weeks compared to babies with gestational age between 32 and 35 weeks. Hemodynamically significant CHD was seen in 4% (*n* = 37) of infants, which was significantly more in children with gestational age <32 weeks.

Majority of the children 75.40% (*n* = 698) had one or more siblings; of them, 8.40% (*n* = 78) had a history of asthma. One-third, 33.10% (*n* = 307), of the children enrolled lived in a smoking environment. Among siblings, 42% (*n* = 389) were in school, and 7% (*n* = 65) had exposure to childcare centers. Multiple births were present in 16.5% (*n* = 153) infants. Most of the babies (81%) (*n* = 757) were enrolled before six months of age. Among them, 63.60% (*n* = 589) and 18.10% (*n* = 168) were less than 3 months and 3–6 months of age, respectively. Only 10.50% (*n* = 98) and 7.50% (*n* = 70) infants were enrolled at age between 7 and 12 months and more than 1 year, respectively.

In comparison to subgroup with a gestational age of 32–35 weeks, children with a gestational age <32 weeks at birth had more incidence of BPD, CHD, family history of asthma, and more siblings with childcare center exposure.

**Figure 1:**
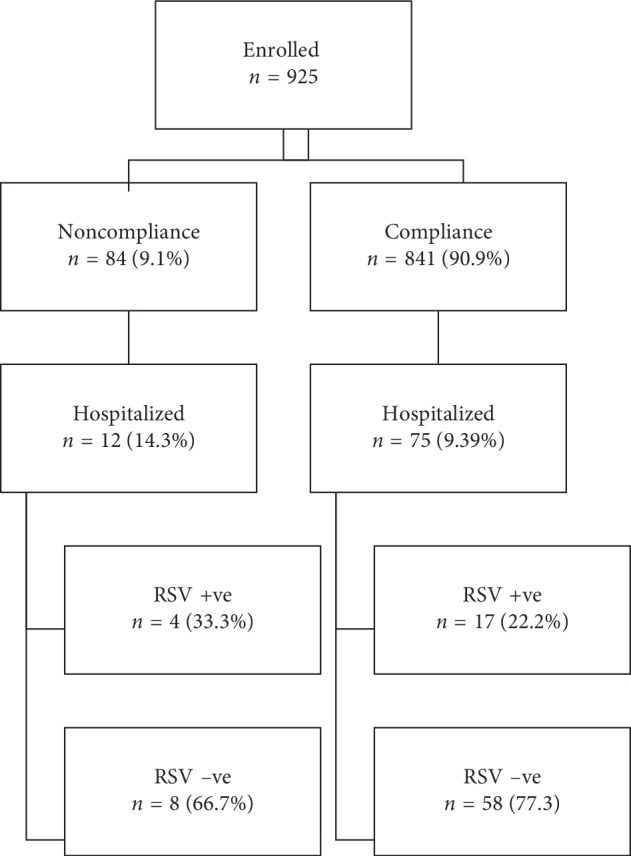
Flow diagram of the study participants and outcomes (gestational age subgroups). RSV, respiratory syncytial virus.

The overall compliance to the protocol with the course of prophylactic treatment with palivizumab was 90.90% (*n* = 841), as shown in Figure 1 and Table 3.

There was no statistically significant difference in the compliance rate between the infants born at <32 weeks and 32–35 weeks of gestation (90.80% and 91%, *p* value 0.84), and only 9% of the children had an interruption in their dosing schedule.

From the enrolled babies, 9.40% (*n* = 87) were hospitalized for acute LRTI. Rate of hospitalization was significantly more in babies with a gestational age <32 weeks compared to the babies with gestational age between 32 and 35 weeks (12.43%, *n* = 51/410, versus 6.99%, *n* = 36/515, *p* value = 0.03).

**Figure 2:**
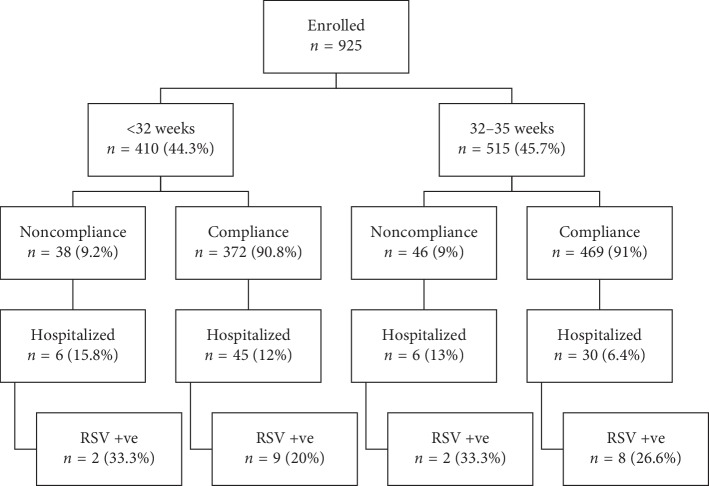
Flow diagram of the study participants and outcomes (compliance subgroups). RSV, respiratory syncytial virus.

Babies from the noncompliant group (*n* = 13/84) had 1.7 times more hospitalization rate compared to compliant subjects (*n* = 75/841) and the unadjusted OR = 1.87 (95% CI: 0.98–3.53, *p* value 0.05). The adjusted odds ratio for gestational age, birth weight, CHD, CLD, and smoke exposure in multivariate regression analysis revealed a high rate of hospitalization in the noncompliant group (OR = 2.32, 95% CI: [1.08–4.12], *p* value 0.04). There is an observed negative correlation between the compliance to palivizumab immunoprophylaxis, gestational age and birth weight, and the rate of hospitalization of at-risk babies. In the group who were compliant to immunoprophylaxis, more babies from <32 weeks group were hospitalized compared to the group with gestational age between 32 and 32 weeks (12.09%, *n* = 45/372, vs 6.39%, *n* = 30/469, *p* value 0.04) (Figure 2 and Table 3).

Babies with hemodynamically significant CHD, CLD, and exposure to smoking environment had a positive correlation on the hospitalization rate.

For children hospitalized due to RSV, the median (IQR) age at the time of first palivizumab dose was 2.1 (1.3) months. Infants with gestational age <32 weeks at birth received immunoprophylaxis later at the median (IQR) age of 2.8 (1.5) months compared to at 1.5 (1.0) months for babies who were 32–35 weeks at birth, but there was wide variation in the age of receiving the first dose (Table 3).

Overall age at hospital admission was 2.35 (1.35) months, and duration of hospital stay was 7.9 (4.6) days. Infants with <32 weeks were older at the time of admission, 3.1 (1.6) months versus 1.6 (1.1) months, and had a longer duration of hospital stay 10.8 (6.0) days versus 5 (3.2) days, when compared to infants with a gestational age of 32–35 weeks, respectively.

Pediatric intensive care unit (PICU) admission rate was 23.5% (*n* = 4/17). Rate of PICU admission in babies who are <32 weeks was about three times higher than babies who were 32–35 weeks at birth (33.33%, *n* = 3/9, versus 12.50%, *n* = 1/8, respectively).

Of the 925 enrolled at-risk cases, 2.27% (*n* = 21) were confirmed RSV positive as detected by RT-PCR. The incidence of positive RSV in the group of <32 weeks of gestation (2.68%, *n* = 11/410) was higher than the 32–35 weeks of the gestation group (1.94%, *n* = 10/515).

Positive RSV infections were lower (2.02%, 17/841) in compliance babies compared to noncompliant subgroups (4.76%, *n* = 4/84), which is about 58% reduction (OR = 2.42, 95% CI: [0.79–7.37], *p* value 0.04).

On the other hand, it was observed that RSV RT-PCR was positive in about 22.66% (*n* = 17/75) in the compliant hospitalized children as compared to 33.33% (*n* = 4/12) in subgroups who were noncompliant to palivizumab (OR = 1.5, 95% CI: [0.41–5.54]).

**Figure 3:**
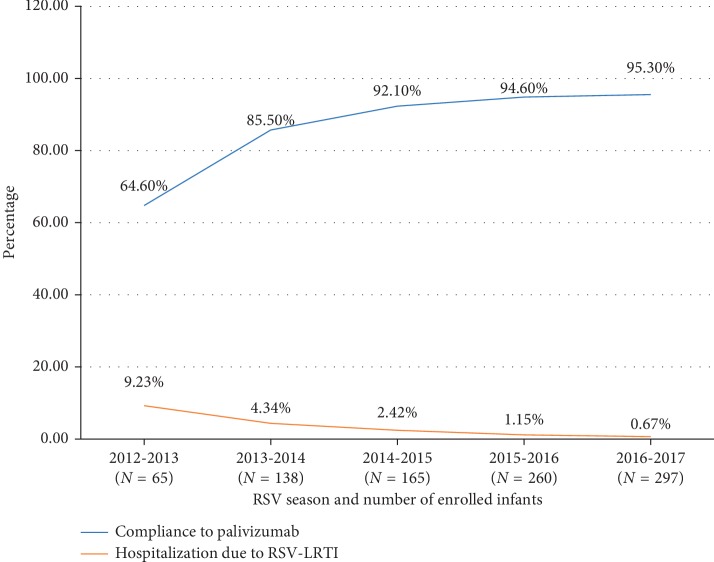
Rates of hospitalization due to RSV infection and compliance to palivizumab across (2012-2013, 2013-2014, 2014-2015, 2015-2016, and 2016-2017) seasons. RSV, respiratory syncytial virus; LRTI, lower respiratory tract infection.

There was a gradual but significant reduction in incidence hospitalization due to RSV-LRTI from 9.23% in the 2012-2013 season to 0.67% in the 2016-2017 season. The reduction in the hospitalization rate could be explained by the increase in the rate of compliance from 64.60% in the 2012-2013 season to 95.3% in the 2016-2017 season, as shown in Figure 3.

## 4. Discussion

The present observational hospital-based study discusses a survey of hospitalizations due to RSV-LRTI infection in children who were eligible for palivizumab prophylaxis. The study covers five RSV seasons (September to March) from 2012 to 2017 at Latifa Women and Children Hospital in UAE. This study is the first of its kind in Dubai, UAE.

This observation gives comprehensive insight on the practice of RSV immunoprophylaxis (palivizumab), compliance with the protocol, and effectiveness in reducing the RSV-LRTI-related hospitalization rate in this part of the Middle East.

The scheduling of palivizumab immunoprophylaxis and infant characteristics included in our study was in accordance with the guideline formulated by the American Academy of Pediatrics and adopted at our institution [19, 21].

We reported a compliance rate to palivizumab prophylaxis of 90.90%, which is significantly higher than that observed in the other published literatures, 85.70% by Abushahin et al. [18], and 81.70% by Borecka and Lauterbach [22] We also registered an increase in compliance across 5 seasons which is comparable to the study quoted above [18]. This can be linked directly to increase awareness campaign and focused approach of care givers, which also observed in other studies [23].

The cumulative RSV infection-related hospitalization accounted for 2.20% of all at-risk babies, which is significantly lower than that observed in the IMpact study group result of 4.80% [17], and the result of a systematic review was conducted in 2016 by Mauskopf et al., where the incidence rates range from 2.30% to 10% [24]. Our data are comparable to other data from the middle east and other countries [7, 18, 25].

The hospitalization rate is significantly higher in the noncompliant group, 15.47% compared to 8.91% in children who were compliant to palivizumab prophylaxis, which corresponded to a significant reduction in incidence of RSV infection (62.40%) in the palivizumab prophylaxis group. Rate of hospitalization was significantly more in babies <32 weeks compared to the other subgroups (32–35 weeks). RSV positivity was lower in compliance babies compared to noncompliant subgroups, which is about 58% reduction in the incidence of RSV infection.

Our result was better than 55% reduction seen in the IMpact study [17]. A 2013 Cochrane meta-analysis of three randomized trials comparing palivizumab with placebo in high-risk infants showed a 50% reduction in RSV-LRTI hospitalizations (from 101 to 50 per 1000) and intensive care unit admissions (from 34 to 17 per 1000) [26]. These results clearly show the protective effect of RSV palivizumab on RSV-LRTI-related hospitalization. In our study, the rate of RSV hospitalization reduced from 9.23% in the year 2012-13 to 0.67% in the year 2016-17. This decrease in hospitalization correlated directly with the increase in compliance to palivizumab (64.60% in 2012-2013 RSV season to 95.30% in 2016-2017 RSV season), which is comparable to the trend observed in other studies [18, 22].

We observed an average length of hospital stay of 7.7 ± 3.67 days, in babies admitted for RSV-related respiratory illness, which is very similar to the available literature [24, 27]. Pediatric intensive care admission rate was 23.50% of our RSV-LRTI hospitalized children, which is slightly higher than 6.50% reported by Viguria et al. [27] but is consistent with the results shown in a systematic review conducted by Mauskopf et al., and these incidences were more in babies of lower gestational age, which is quite similar to our result [24]. There was no death observed in our study.

The Japanese survey of pediatric ward hospitalization due to RSV infection after the introduction of palivizumab to high-risk infants showed that 8163 children were admitted to participating hospitals. Of that, 811 (9.93%) children had confirmed RSV-LRTI, with a mean gestational age of those at birth was 38 weeks. This study found that the appropriate use of palivizumab could reduce the hospitalization rate by 72% in infants and young children with a gestational age of 33–35 weeks [28].

In our study, we observed good tolerance to palivizumab injection, and no adverse effects were observed to the therapy. Our observation is consistent with other reports on palivizumab safety profiles [29].

Our study is the first of its kind in Dubai, United Arab Emirates. The data collection process is robust. This research gave us baseline data regarding RSV infection and the effect of RSV immunoprophylaxis in the region.

The main limitation of this study is that it is a retrospective analysis. Retrospective observational studies limit the generalizability of the results. So, a randomized trial is needed to determine the efficacy of RSV prophylaxis in specific high-risk infants. We were not able to include at-risk children who were admitted to other hospitals or migrated out of UAE and have RSV-LRTI.

## 5. Conclusion

Our study demonstrated that RSV immunoprophylaxis with palivizumab in high-risk children leads to decrease in the incidence of RSV-related respiratory infection and related hospitalization. With increased awareness and effective implementation, high compliance to RSV immunoprophylaxis could be achieved.

## Figures and Tables

**Table 1:** Inclusion criteria for RSV immunoprophylaxis (palivizumab).

Inclusion criteria	Age at the start of RSV season
Gestational age ≤28 weeks	≤12 months
Gestational age ≥29 to <32 weeks	≤6 months
Gestational age ≥32 to <35 weeks	≤6 months and RSV high-risk score of ≥49
Hemodynamically significant CHD, CCF, pulmonary hypertension, etc.	≤6 months
CLD who required medical therapy or required medical treatment within preceding 6 months before starting RSV season	≤24 months
Congenital anomaly of airways or neuromuscular diseases that compromise handling of respiratory secretions	≤24 months
Cystic fibrosis, immunocompromised	≤24 months (case by case basis)

RSV, respiratory syncytial virus; CHD, congenital heart disease; CCF; congestive cardiac failure.

**Table 2:** Babies' characteristics during a total of 5 seasons.

Variables	Total babies (*N* = 925)	<32 weeks (*N* = 410)	32–35 weeks (*N* = 515)	*p* value
Gender, *N* (%)				
Female	501 (54.10%)	245 (59.80%)	256 (49.70%)	0.38
Male	424 (46.60%)	165 (40.20%)	259 (50.2%)	
Gestational age in weeks, median (IQR)	30.28 (4.1)	27.52 (4.3)	33.04 (4.2)	0.35
Female	30.60 (3.8)	27.90 (4.1)	33.3 (3.95)	
Male	29.96 (4.4)	27.12 (4.5)	32.8 (4.45)	
Birth weight, g median (IQR)	1460.6 (410.2)	1100.8 (383.8)	1820.5 (397)	0.42
Female	1482.5 (398.1)	1116.5 (365.1)	1848.5 (431)	
Male	1438.7 (422.3)	1084 (402.5)	1792 (442)	
Age at enrollment, *N* (%)			390 (75.70%)	0.42
≤3 months	589 (63.60%)	199 (48.50%)	50 (9.70%)	
4–6 months	168 (18.10%)	118 (22.90%)	29 (5.60%)	
7–12 months	98 (10.50%)	69 (16.80%)	7 (1.30%)	
>12 months	70 (7.50%)	63 (15.30%)		
Multiple birth, *N* (%)	153 (16.50%)	105 (25.60%)	48 (9.30%)	0.15
Chronic lungs disease, *N* (%)	180 (19.40%)	122 (29.70%)	58 (11.20%)	0.09
Congenital heart disease, *N* (%)	37 (4%)	30 (7.30%)	7 (1.30%)	0.04
Presence of siblings, *N* (%)	698 (75.40%)	333 (81.20%)	365 (70.80%)	0.71
Siblings in school, *N* (%)	389 (42%)	140 (34.10%)	249 (48.30%)	0.23
Parental asthma, *N* (%)	135 (14.50%)	76 (18.50%)	59 (11.40%)	0.85
Sibling history of asthma, *N* (%)	78 (8.43%)	44 (10.73%)	34 (6.60%)	0.62
Mother's age, years median (IQR)	30.5 (9)	31.6 (9.4)	29.4 (8.6)	0.36
Childcare center exposure, *N* (%)	65 (7%)	49 (11.90%)	16 (3.10%)	0.18
Smoke exposure, *N* (%)	307 (33.10%)	157 (38.20%)	150 (29.10%)	0.29

RSV, respiratory syncytial virus; IQR, interquartile range.

**Table 3:** RSV-related respiratory disease characteristics of hospitalized infants during a total of 5 seasons.

Variables	Total babies (*N* = 925)	<32 weeks (*N* = 410)	32–35 weeks (*N* = 515)	*p* value
Compliance (received prophylaxis as per guidelines), *N* (%)	841 (90.90%)	372 (90.80%)	469 (91%)	0.84
Babies hospitalized (from compliance babies), *N* (%)	75/841 (8.91%)	45/372 (12.01%)	30/469 (6.39%)	0.04
RSV positive among compliance babies, *N* (%)	17/841 (2.02%)	9/372 (2.41%)	8/469 (1.70%)	0.46
RSV positive among hospitalized babies (compliance babies), *N* (%)	17/75 (22.66%)	9/45 (20%)	8/30 (26.66%)	0.50
Total hospitalized baby (from at-risk population), *N* (%)	87/925 (9.40%)	51/410 (12.43%)	36/515 (6.99%)	0.03
RSV positive in all at risk babies, *N* (%)	21/925 (2.27%)	11/410 (2.68%)	10/515 (1.94%)	0.45
RSV positive among at risk hospitalized babies, *N* (%)	21/87 (24.13%)	11/51 (21.56%)	10/36 (27.77%)	0.50
Babies hospitalized (noncompliance babies), *N* (%)	12/84 (14.28%)	6/38 (15.78%)	6/46 (13.04%)	0.50
RSV positivity in babies not received palivizumab, *N* (%)	4/84 (4.76%)	2/38 (5.26%)	2/46 (4.3%)	0.80
RSV-related length of stay, days median (IQR)	7.9 (4.6)	10.8 (6.0)	5 (3.2)	0.15
RSV-related PICU admission, *N* (%)	4/17 (23.50%)	3/9 (33.30%)	1/8 (12.50%)	0.32
Age at the 1st dose of palivizumab for RSV hospitalized babies, months median (IQR)	2.1 (1.3)	2.8 (1.5)	1.5 (1.0)	0.26
Age at hospital admission, months median (IQR)	2.35 (1.35)	3.1 (1.6)	1.6 (1.1)	0.30

RSV, respiratory syncytial virus; IQR, interquartile range; PICU, pediatric intensive care unit.

## Data Availability

The data used to support the findings of this study are available from the corresponding author upon request.
